# Modification on Direct Agglutination Antigen Preparation for Simplified Sero-Diagnosis of Human and Canine Visceral Leishmaniasis

**Published:** 2015

**Authors:** Mehdi MOHEBALI, Behnaz AKHOUNDI, Zahra KAKOOEI, Zabih ZAREI, Sorour CHAREHDAR, Soheila MOLAEI

**Affiliations:** 1 *Dept. of Medical Parasitology and Mycology, School of Public Health, Tehran University of Medical Sciences, Tehran, Iran*; 2 *Center for Research of Endemic Parasites of Iran (CREPI), Tehran University of Medical Sciences, Tehran, Iran*; 3 *Meshkin Shahr Research Station, School of Public Health, Tehran University of Medical Sciences, Meshkin Shahr, Iran*; 4 *Meshkin Shahr Health Centre, Ardabil University of Medical Sciences, Meshkin Shahr, Iran*

**Keywords:** Direct agglutination test, Antigen modification, Visceral leishmaniasis

## Abstract

***Background:*** Visceral leishmaniasis is systematic serous parasitic disease with public health importance. Zoonotic form of visceral leishmaniasis is wide spread in Mediterranean basin and South America regions. Direct agglutination test (DAT) is an accurate, reliable and non-expensive serological test for the diagnosis of visceral leishmaniasis in human and canines but the antigen preparation involves some limitations. This study aimed to compare the conventional production of DAT antigen with our modified DAT antigen and then assessed on human and dog pooled sera.

***Methods:*** Conventional DAT antigen has been prepared at the School of Public Health, Tehran University of Medical Sciences and some modifications were carried out on it, which named as modified DAT antigen. Three positive and one negative human and dog pooled serum were separately used for the comparison of modified DAT with conventional DAT antigen batches with one-month interval for a period of 9 months.

***Results:*** A good concordance was observed between modified DAT compared to conventional DAT antigens for the detection of visceral leishmaniasis on human (100%) and dog (94.4%) pooled sera, respectively.

***Conclusion:*** Since the modified DAT antigen could be reduced the preparation time from 3 days to several hours and a good degree of agreement was found between modified DAT and convention DAT antigen batches, it can be used as a simple and easy tool for screening and serodiagnosis of human and canine *L. infantum* infection.

## Introduction

Visceral leishmaniasis (VL) is a potentially fatal protozoan infection that is endemic in some parts of Iran (1). Majority of human VL cases in Iran have been reported from rural areas of the northwestern and southern regions, where health facilities are not well established and visceral leishmaniasis often co-exists with brucellosis, tuberculosis and other infectious diseases (2- 5). Domestic dogs (*Canis familiaris*) are principal VL reservoir hosts that can carry either *L. infantum/chagasi* (6). In addition, canine visceral leishmaniasis (CVL) is endemic in northwestern and southern Iran, where its prevalence ranges from 14.2% to 17.4% (7- 9).

Parasitological, serological and molecular methods are used for the diagnosis of human VL. Microscopical examinations were performed on bone marrow materials as well as spleen samples, but these methods are invasive (6, 10).

 Although the IFAT and ELISA are two important serological methods for the detection of human VL but these methods are required to specific materials and equipments. Other serological methods, such as the latex agglutination test (KAtex^®^) and recombinant antigens dipstick (rK39, rK26, rKe16) have limitations, such as low specificity in Sudanese subjects in the absence of clinical visceral leishmaniasis (10-13). 

Molecular methods are sensitive but have high variations of specificity, require sophisticated equipments and to identify of *Leishmania *species (1).

Based on previous studies, direct agglutination test (DAT) is an easily performed, highly sensitive, specific, reliable and cost-effective technique for the diagnosis and sero-epidemiological study of VL in humans and dogs across different geographical regions. With this method, a small amount of serum or plasma specimens, and even a drop of dried blood on filter paper is taken from the tip of the finger is possible (1, 2, 4, 5, 14, 15).

As conventional DAT antigen has been prepared at the School of Public Health, TUMS needs to long times for antigen production. Therefore, this study aimed to reduce the duration times of DAT production from three days to 5 hours.

## Materials and Methods

The principal steps for making DAT antigen were the mass production of promastigotes of the Iranian strain of *L. infantum* [MCA-N/IR/07/Moheb-gh. (GenBank accession no. FJ555210)] in RPMI 1640 medium (Biosera, South America) containing 10% fetal calf serum (Biosera, South America), trypsinization of the parasites, staining with Coomassie brilliant blue R-250 (Sigma, USA) and fixing with 2% formaldehyde (2, 4, 5, 16). On the other hand, for preparation of DAT antigen based on Gomez-Ochoa method the concentration of the culture was determined by counting the promastigotes in a Neubar chamber and standardizing the concentration at 10^9^ promastigotes/ml; 0.2 g trypsin (at a 1:250 dilution with γ-irradiated porcine pancreas; Panreac) was added to the culture, which was maintained at 37°C for 45 min. After this time, the culture was placed in a frozen water bath to stop trypsinization. Then, 130 μl of formalin (37 % *P*/*P* ethanol stabilized; Panreac) to each 200 ml culture media were added. The culture was stirred gently for 1 h to fix the promastigotes properly. For harvesting, the culture was centrifuged (in 50 ml Falcon tubes at 2000 × *g* for 10 min) two times in order to concentrate the promastigotes and one time with citrate saline solution to remove excess formaldehyde. Finally, the pellet was dissolved in 25 ml of citrate saline solution. 

To stain the promastigotes, we used Coomassie brilliant blue (R-250, Merck) diluted to 0.5% (w/v), we added 25 ml of this solution to the fixed promastigotes, which produced a final concentration of 0.25%. After stirring the mixture gently for 90 min, we harvested the promastigotes by centrifuging the mixture three times at 2000 *g* for 10 min, washing it each time in citrate saline solution. To store this new antigen produced, we dissolved the pellet in citrate saline solution with 0.4% formalin at a concentration of 50 × 10^6^ promastigotes/ml and then preserved the solution, protected from light, kept at 4°C. It is important to emphasize that the entire procedure was performed at room temperature and that the process was not carried out under sterile conditions. The final antigen product was not contaminated since the original culture was sterile and the formaldehyde, which was added 45 min after processing, prevented any subsequent contamination (17).

All batches of DAT antigens were produced in the School of Public Health, TUMS. Both productions of conventional DAT and modified DAT antigen batches were compared in Table 1.

**Table 1:** Different steps of conventional DAT antigen production in comparison with the modified antigen

**Conventional method**	**Modified method**
Need to 5 days culture	Need to 5 days culture (number of promastigotes 1×10^9^ /ml)
Centrifugation and washing (3times, refrigerated centrifuge)	Not required
Adding trypsin 0.4% to precipitate	Adding trypsin 0.2% to total culture
Centrifugation and washing (3times, refrigerated centrifuge)	Not required
Counting by Neubauer slide chamber	Not required
Fixation (Adding Formaldehyde 2%) set the number of promastigotes to 2×10^8^ /ml	Fixation (Adding 130 µl commercial Formaldehyde to each 200 ml culture media)
Centrifugation and washing 3 times (refrigerated centrifuge)	Centrifugation and washing 2 times ( possibility using non-refrigerated centrifuge) and the preparation of 25 ml suspension
Staining with Coomassie brilliant blue 0.02% for 24-48 hours	Staining with Coomassie brilliant blue 0.5% for 90 minutes
Centrifugation and washing 3 times (refrigerated centrifuge) and the concentration adjusted	Centrifugation and washing 2 times (possibility using non-refrigerated centrifuge) and the concentration adjusted

For preparation of pooled human and dog sera with different criteria, 30 samples for each positive pooled sera and negative pooled sera were collected from endemic and non-endemic areas of VL, respectively. The titers of positive low pooled serum were in a rang of 1:400 to 1:3200 for human samples and a grade of 1:40 to 1:320 for dog samples, the titers of medium pooled serum (intermediate pooled serum) was 1:6400 to 1:51200 for human and for dog with 1:640 to 1:10240. In addition, the titers of high-pooled serum from human and dog sera were with a grade of 1:102400 and 1:20480, respectively. However, total serum samples in each group were 5-10.

The pooled human sera were diluted over a range of 1:10 to 1:102400 with normal saline (0.9% NaCl) containing 0.2% gelatin and 0.78% 2-mercaptoethanol (2-ME) (Sigma Lot No. 45H0508). The pooled canine sera were diluted from 1:10 to 1:20480 with the same diluents, but 1.56% 2-ME were added to the V-shape microtiter plates. The dog sera plates were incubated for 1 h at 37 ^o ^C.

After adding the respective antigens, the microplates were manually shaken for 1 min. Following 12-18 h incubation under ambient temperatures and moist conditions, the results were assessed. The highest dilution showing agglutination was considered as final titer. 

The new DAT antigen was evaluated on human and dog positive and negative pooled sera, compared with the conventional DAT antigen and were repeated with the same conditions with one-month interval for a period of 9 months.

Statistical analyses were conducted using SPSS software version 13.5 (SPSS Inc., Chicago, IL, USA), with a probability (*P*) value of less than 0.05 considered to be statistically significant. The degree of concordance was determined by calculating the total number of positive samples with both antigens plus the total number of negative samples with both antigens divided by the total number of samples, using a 95% confidence interval. A value of 0.21-0.60 represents a fair to moderate agreement, a value of 0.61-0.80 represents a substantial concordance, and a value ≥ 0.81 represents almost perfect agreement (18). 

## Results

All prepared pooled serum samples were tested with the conventional DAT antigen and with the modified DAT antigen methods. The modified DAT results showed 100% sensitivity (27 pooled positives and no negatives out of 9 samples determined to be positive by parasitological and conventional DAT methods and tested with one month interval for a period of 9 months, 100% specificity (9 negatives pooled sera out of 9 samples determined to be negative by conventional DAT method with one month interval for a period of 9 months) in human and dog sera. In addition, a positive predictive value of 100% was found as well as a negative predictive value of 100%. The cut off titer was established as 1:800 to obtain identical titers for both procedures. Moreover, we tested the same serum samples again every month to obtain the durability of the modified DAT antigen. The antigen remains durable for 9 months, a result similar to that for the conventional DAT antigen. Moreover, reproducibility rates of modified antigen on human and dog samples compared with conventional antigen were found 94.4% and 91.7%, respectively. Stability of both antigens was estimated for at least 9 months. 

By means of new antigen, all 27 positive pooled sera showed the same titer in anti-*Leishmania* antibodies in comparison with conventional DAT antigen while in dog sera 2 out of 27 positive pooled sera had one fold of anti-*Leishmania* antibodies reduction. 

A good degree of concordance was observed between modified DAT and convention DAT antigens was observed on human (100%) and dog (94.4%) pooled sera, respectively by kappa analysis (*P*<0.05). 

## Discussion

Visceral leishmaniasis is a serious disease that is fatal in about half a million people worldwide are infected annually. Therefore, early diagnosis and early treatment is very important. Although, bone marrow aspiration is a golden standard for the diagnosis of visceral leishmaniasis but this is an invasive procedure. Serological tests as none invasive methods with high sensitivity and specificity rates can be replaced of parasitological procedures (6). Among different methods, the direct agglutination test (DAT) is a simple, valid, and reliable in the field (4, 15). 

Since VL is related to poverty and it occurs mainly in areas where health services are poorly developed thus, research priority has been focused on the development of a simple, cheap, accurate and reliable diagnostic test for the diagnosis and sero-epidemiological of the disease (19). The DAT has been introduced and developed for use under field conditions (20-22) but the most problem of the test depends on antigen preparation (23). Gomez-Ochoa et al. in 2003 reduced the duration of DAT antigen preparation from 3 days to a few hours, with 1×10^9^ per ml of culture medium (about a billion), and small changes in the procedure which was called Easy-DAT. Sensitivity and specificity of this antigen in comparison with conventional DAT antigen on dog sera samples were 100% and 98.7%, respectively. Although, their study was performed by homologous isolate, due to lack of visceral patient samples they only used dog sera. Gomez-Ochoa et al. used a cut off 1:800 and were evaluated Easy-DAT monthly during 6 months (17). 

Our Modified DAT antigen method shows the same sensitivity, specificity, and durability compared to the conventional DAT antigen method but has the additional advantages of cost reduction in antigen production, standardization of some specific materials such as trypsin and formalin needed to the number of promastigotes and reduction of the antigen elaboration time to only 5 hours. This modified DAT antigen procedure makes antigen production easier, faster and thus has the diagnostic test more accessible in under-developed endemic areas of human and canine VL.

In the present study, we used positive and negative pooled sera from endemic and non-endemic areas in order to reduce the influence of genetic differences between humans and dogs MHC system, which is one of the most important confounding factors in different geographical areas (10).

As was mentioned, the reducing of the stage numbers of new DAT antigen preparation (from homologous *L. infantum* isolate) consist of centrifugation and washing, fixation and staining reduce of preparation time from 3 days to several hours. Therefore, this method is simple, practical and available in our country.

## Conclusion

Since the modified DAT antigen could be reduced the preparation time from 3 days to several hours and a good degree of agreement was found between modified DAT and convention DAT antigen batches, it can be used as a simple and easy tool for screening and serodiagnosis of human and canine *L. infantum* infection.
